# Neurocognitive functioning in adults with neurofibromatosis type 1- a nationwide population-based study

**DOI:** 10.1186/s13023-024-03454-w

**Published:** 2024-11-28

**Authors:** Karoline Doser, Jens Richardt Møllegaard Jepsen, Line Kenborg, Kamilla Woznica Miskowiak, Vanna Albieri, Susanne Oksbjerg Dalton, Anja Krøyer, Hanne Hove, John R. Østergaard, Christoffer Johansen, Sven Asger Sørensen, John Mulvihill, Jeanette Falck Winther, Pernille Envold Bidstrup

**Affiliations:** 1 Psychological Aspects of Cancer, Danish Cancer Institute, Strandboulevarden 49, Copenhagen, 2100 Denmark; 2 Center for Clinical Intervention and Center for Neuropsychiatric Schizophrenia Research, Capital Region Copenhagen, University of Copenhagen, Copenhagen, Denmark; 3 Child and Adolescent Mental Health Centre, Mental Health Services Capital Region Copenhagen, University of Copenhagen, Copenhagen, Denmark; 4 Childhood Cancer Research Group, Danish Cancer Institute, Copenhagen, Denmark; 5 Copenhagen Affective Disorder Research Centre (CADIC), Psychiatric Centre Copenhagen, Copenhagen University Hospital Rigshospitalet, Copenhagen, Denmark; 6 Department of Psychology, University of Copenhagen, Copenhagen, Denmark; 7 Statistics and Data Analysis, Danish Cancer Institute, Copenhagen, Denmark; 8 Cancer Survivorship, Danish Cancer Institute, Copenhagen, Denmark; 9 Department of Clinical Oncology & Palliative Care, Zealand University Hospital, Næstved, Denmark; 10 Institute of Clinical Medicine, Faculty of Health, University of Copenhagen, Copenhagen, Denmark; 11 Centre for Rare Diseases, Copenhagen University Hospital, Rigshospitalet, Copenhagen, Denmark; 12 The RAREDIS Database, National Rare Disease Database, Copenhagen, Denmark; 13 Center for Rare Disease, Aarhus University Hospital, Aarhus, Denmark; 14 CASTLE Cancer Late Effect Research, Oncology Clinic, Copenhagen University Hospital Rigshospitalet, Copenhagen, Denmark; 15 Department of Pediatrics, University of Oklahoma, Oklahoma City, USA; 16 Department of Clinical Medicine, Faculty of Health, Aarhus University and University Hospital, Aarhus, Denmark

**Keywords:** Neurofibromatosis type 1, Adults, Neurocognitive functioning, Executive functioning

## Abstract

**Background:**

Neurofibromatosis type 1 (NF1) is a genetic condition characterized by various somatic manifestations and cognitive impairments, but the latter are sparsely described in adults. This study aimed at characterizing potential impairments of neurocognitive functions using neuropsychological tests as well as a self-report questionnaire.

**Methods:**

In a nationwide, population-based study including 103 adults with NF1 and 38 age- and gender-matched NF1-free comparisons, we used a comprehensive neurocognitive test battery to assess intelligence and visual short-term memory, immediate visuospatial recall, reaction time, sustained attention, motor speed, planning, planning time, working memory as well as multitasking and a questionnaire to assess executive functions. Descriptive statistics, multivariate analysis of variance (MANOVA), and general linear models with repeated measure analysis of variance (ANOVA) were used.

**Results:**

We observed a statistically significant difference in overall performance-based cognitive functioning. Adults with NF1 showed significant, moderate-to-severe impairments in intelligence, visual short-term memory, immediate visuospatial recall, sustained attention (*p* < 0.0001–0.002), and some executive functions (*p* = 0.008 − 0.001), whereas other cognitive functions (multitasking, reaction time, motor speed, spatial working memory, planning time, and planning efficacy as well as some self-reported executive functions) were unimpaired.

**Conclusions:**

This is the first study with a population-based sample of persons with NF1 and the results show impairments of intelligence and other cognitive functions. The pattern of both significant cognitive impairments and non-significantly different cognitive functions suggests a cognitive profile of selective rather than generalized cognitive deficits in NF1.

**Supplementary Information:**

The online version contains supplementary material available at 10.1186/s13023-024-03454-w.

## Background

Persons with the autosomal dominant genetic disorder, neurofibromatosis type 1 (NF1) are at increased risk of somatic [1], psychiatric [2] and cognitive implications [3, 4]. Knowledge of the cognitive impairments associated with NF1 is essential for understanding the implications of NF1 on everyday adult life functioning. A broad range of cognitive deficits has been described in up to 81% of persons with NF1 from childhood to adult life [5, 6]. In children, potential impairment has in a number of studies been shown in visuospatial, attention, executive, motor, and language functions [4], and to some degree in memory [3]. However, in adults with NF1, the nature and severity of neurocognitive deficits is poorly described, with only few small case-control studies (*N* = 5–48) of adults or mixed samples of children, adolescents, and adults [5, 7–13].

With respect to intelligence, significantly lower verbal and performance IQ were observed in adults with NF1 compared to their age- and gender-matched peers (NF1: mean verbal IQ = 85, SD = 16.6; controls: mean verbal IQ = 99, SD = 12.9, large effect size; NF1: mean performance IQ = 87, SD = 15.3; controls: mean performance IQ = 98, SD = 19.6,, moderate effect size) [8]. In adults with NF1, impairments of immediate and delayed verbal memory [13, 14], working memory [9, 10] as well as visual memory [5, 10, 13] were observed. No indication of impaired motor functioning or motor skill learning has been found in adults with NF1 [5, 8], however, only two studies have examined this domain.

While 30–50% of children with NF1 aged 6–16 years have Attention Deficit Hyperactivity Disorder (ADHD) [6, 15, 16], the prevalence of ADHD has not been estimated in adults with NF1. Zöller et al. (1997) found statistically significant and moderate impairments of attention in adults with NF1 compared with age-, education-, and gender-matched controls [5]. However, Castricum et al. (2022) did not find significantly reduced alertness or sustained attention in a study including adults with NF1 and age- and gender-matched peers [8].

Reaction time has not yet been investigated in adults with NF1. In a small study of adolescents with NF1 and age- and gender-matched healthy controls, those with NF1 had slower mean reaction time with a very large effect size [17]. Similarly, Ferner et al. (1996) found significantly slower mean reaction times in their mixed age-group sample of 103 persons with NF1 (age range, 6–75 years) compared to age- and gender-matched controls [14]. Due to the mixed age sample reaction time functioning in adults with NF1 remains unclear. Studies have demonstrated significant impairments in executive functions in adolescents with NF1, including inhibitory control and working memory [17] and mental flexibility in psychiatrically healthy adults with NF1 [5]. However results are mixed and several studies found no significant differences in executive functions between adults with and without NF1 [10, 12, 13].

Further characterization of potential impairments of intelligence and neurocognitive functions across several domains of adults with NF1 is warranted. We combined objective, performance-based tests with a self-reported questionnaire assessment and established a large-scale population-based cohort of adults with NF1 and NF1-free, age- and gender-matched comparisons from the unique Danish registers. The purpose was to characterize potential differences in intelligence and specific neurocognitive functions across several cognitive domains, according to NF1 status. We hypothesized deficits in intelligence and cognitive impairments across all domains assessed, i.e., a generalized profile of significant cognitive impairments.

## Methods

**Fig. 1:**
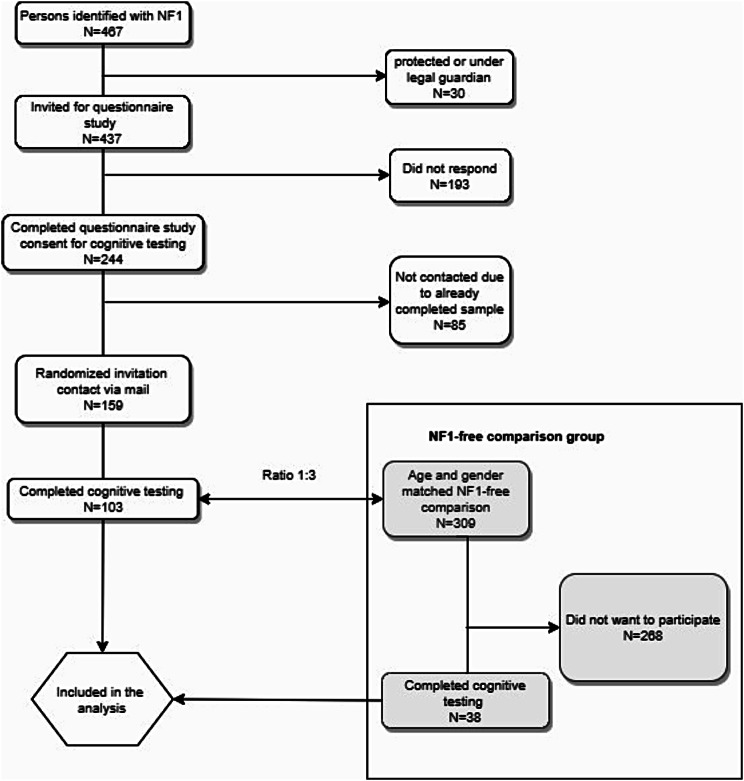
Identification of NF1-group (*N* = 103) and age- and gender-matched NF1-free comparison group (*N* = 38)

In this population-based study, we included adults with NF1 and age- and gender- matched population-based NF1-free adults. The study was nested in a previous nationwide, cross-sectional questionnaire study (response rate: 56%) including 244 adults aged ≥ 18 years diagnosed with NF1 at one of the only two National Centers of Rare Diseases located at Copenhagen University Hospital, Rigshospitalet, and at Aarhus University Hospital between 1977 and 2016 [18]. Exclusion criteria included inability to read Danish and inability to provide informed consent. A total of 159 adults with NF1 were invited for the current sub-study during May 2018 and July 2019 and of these,103 adults with NF1 provided written consent (response rate: 65%) (Fig. 1).

From the Danish Civil Registration System, we invited gender- and age-matched (1:3) population-based adults without NF1 and included 38 in the NF1-free control group (response rate: 13%). The participants were contacted to arrange the test appointment, which lasted 2.5–3 h, at an appropriate location (i.e. a suitable quiet room with no distractions) at home or, in a few cases at a local counseling center. The tests were performed by 10 psychology students, who initially underwent a four-day training program including a final assessment of alignment with the test manual describing all test procedures. Permissions were obtained from the Institutional Review Board at the Danish Cancer Intitute, the local Institutional Review Board, and the Research Board of the Department of Defense US Army under Award No. W81XWH-14-1-0054. The data collection procedure was approved by the Danish Health Data Authority. Further, the NF1-program was registered at the Danish Cancer Society Research Centers archive (2018-DCRC-0012).

### Cognitive functioning

An overview of all measures is included in supplementary Table A. Estimated intelligence (estimated FSIQ) was assessed with an abbreviated version of the Wechsler Adult Intelligence Scale, fourth edition (WAIS-IV) [19] including the following subtests: Vocabulary, Similarities, Block Design, and Matrix Reasoning (supplementary material A). We used selected tests from the Cambridge Neuropsychological Test Automated Battery (CANTAB Connect, tablet version) [20, 21] to assess visual short-term memory (or alternatively interpreted as reflecting visuospatial working memory capacity [22]) (Spatial Span (SSP, forward span length)), multitasking (Multitasking Test (MTT, incongruency cost)), reaction time (Reaction Time (RTI, median simple reaction time and median five choice reaction time)), motor speed (Movement Time (RTI, median simple movement time and median five choice movement time)), spatial working memory (Spatial Working Memory (SWM, total errors)), planning and planning time (One-touch Stocking of Cambridge (OTS, problem solved on first trial, median latency on first trail)), and sustained attention (Rapid Visual Information Processing (RVP, A´)) (Supplementary material A). Immediate visuospatial recall was assessed with the Rey’s Complex Figure Task (RCFT) with a 3-minute time gap [23] (supplementary material A). To assess executive functions in everyday life, we used the Danish self-report version of the Behavior Rating Inventory of Executive Function – Adult version (BRIEF-A) [24] which remains to be validated in Danish (supplementary material A). KD and JRMJ independently scored the test performance of ten randomly selected participants, and the two inter-scorer reliability estimates were excellent with an intra-class correlation coefficient of 0.949 for the WAIS-IV verbal IQ and 0.926 for the RCFT recall score.

### Socio-demographic and clinical characteristics

Socio-demographic and clinical characteristics (disease severity, disease visibility) were obtained from a self-reported questionnaire. Socio-demographic characteristics included age, gender, highest attained education (grouped according to the International Standard Classification of Education (ISCED-2011): short (≤ 9 years), medium (10–12 years), and long (> 12 years)) [18], employment status (employed, unemployed, social transfer payments), living status (alone, with spouse, with parents, shared home or institution), and partner status (having partner, yes/no).

#### Disease severity

We developed a self-report version of the Riccardi scale [25] to assess NF1 disease severity [18]. The original Riccardi scale was developed for physicians to evaluate severity (level 1–4) through a checklist including a combination of clinical features, symptoms and their impact on well-being. For our modified version, we developed 13 binary self-report items (yes or no) which corresponds to the four severity levels as mild (level 1 + 2), moderate (level 3), or severe (level 4).

#### Disease visibility

We developed a self-report version of the Ablon scale [26] assessing visibility of NF1 [18]. The original Ablon scale was developed for physicians to evaluate visibility (grade 1–3) through features including e.g. café-au lait spots, tumors on neck or face, or noticeable limp. We developed six self-report binary items (yes or no) which correspond to the three levels as grade 1 (mild), grade 2 (moderate), and grade 3 (severe). Both the self-reported disease severity and visibility scales remains to be validated.

### Statistical analyses

To characterize the NF1 and NF1-free comparison groups, descriptive statistics were conducted for demographic and disease-related factors. Independent-samples *t-*tests and Fishers exact tests were conducted for normally distributed data and nominal data, respectively, to determine any potential difference in demographic variables (age, gender) between NF1 and NF1-free persons.

The raw scores for performance-based and self-reported outcomes were converted to z-scores according to the control group’s mean raw scores and standard deviations to establish a z-score mean of 0 and a standard deviation of 1.0 as a reference for the control group. For selected variables the z-score transformations were reversed to ensure that higher z-scores indicated better performance. For reaction time, we used the re-standardized mean z-score of the median simple reaction time z-score and the median five choice reaction time z-score. Similarly, for motor speed, we used the re-standardized mean z-score of the median simple movement time z-score and the median five choice movement time z-score. Assumptions about normal distributions were inspected in box plots, and tests for homogeneity were performed. Neuropsychological test outcome scores were missing in only two NF1-free controls (multitasking, *n* = 1, sustained attention *n* = 1, 2.7%) and in four persons with NF1 (estimated intelligence (*n* = 1, 1%), immediate visuospatial recall (*n* = 2, 1.9%), and sustained attention, (*n* = 1, 1%). Missing scores were replaced by the mean raw scores of the relevant group to allow analysis of the total sample. In multivariate analysis of variance (MANOVA), the z-values of all performance-based outcomes and self-reported outcomes were examined according to NF1 status, followed by a series of ANOVA. A sensitivity analysis was performed, repeating the main analyses while excluding three NF1-free persons who were considered outliers, defined as a z-score <–2.5 in at least two of the cognitive outcome variables. A Bonferroni-corrected alpha level of 0.005 was defined, in view of the number of statistical analyses.

To characterize the cognitive profile and the executive functioning profile as flat or significantly jagged in the group of persons with NF1, a general linear model with repeated measure ANOVA and a Greenhouse-Geisser correction was conducted on cognitive functions z-scores derived from the performance-based tests and separately in an explorative analysis on self-reported executive functions scales z-scores. The z-scores were within-subject outcomes and NF1 status a between-subjects factor. In case of a significant profile, the cognitive profile of persons with NF1 was further characterized using paired-samples *t-*tests.

As described, highly correlated test outcome scores for reaction time and motor speed, respectively, were merged to avoid shared variance of cognitive functions scores in the analysis [RTI; simple median reaction time and median five-choice reaction time (rho = 0.810, *p* < 0.0001)) and motor speed (RTI; median simple movement time and median five-choice movement time (rho = 0.939, *p* < 0.0001)] (all variable outcomes, see supplementary Table B). Statistical analyses were performed with SPSS Statistics 26 software.

## Results

### Socio-demographic characteristics of the NF1 and NF1-free group

The study cohort comprised 103 persons with NF1 and 38 persons without NF1 (Table 1).

**Table 1:** Characteristics of the NF1 cohort (*N* = 103) and NF1-free comparison cohort (*N* = 38)

Characteristic		NF1 *N* = 103 *N* (%)	NF1-free *N* = 38 *N* (%)	*P*-value^3^
**Age**,** mean (SD)**		43.28 (15.9)	45.3 (17.3)	0.516
**Gender**	Male	52 (50.5)	17 (44.7)	0.57
	Female	51 (49.5)	21 (55.3)	
**Severity**	Mild	34 (33.0)	--	
	Moderate	26 (25.2)	--	
	Severe	43 (41.7)	--	
**Visibility**	Mild	14 (13.6)	--	
	Moderate	21 (20.4)	--	
	Severe	68 (66.0)	--	
**Highest attained education** ^**1**^	Short or medium^**2**^	56 (54.3)	15 (39.5)	0.03
	Long	35 (34.0)	23 (60.5)	
	Missing	12 (11.7)	--	
**Employment status**	Employed	14 (13.6)	27 (71.1)	< 0.0001
	Unemployed	44 (42.7)	4 (10.5)	
	Social transfer	41 (39.8)	7 (18.4)	
	Missing	4 (3.9)	--	
**Living status**	Living alone	46 (44.7)	6 (15.8)	< 0.0001
	Living with spouse	43 (41.7)	28 (73.7)	
	Living with parent	9 (8.7)	--	
	Living in shared home	--	4 (10.5)	
	Missing	5 (4.9)	--	
**Cohabitation status**	Having partner	49 (47.6)	31 (81.6)	0.0003
	Having no partner or missing^**2**^	54 (52.4)	7 (18.4)	

NF1: Neurofibromatosis type 1; SD: Standard deviation

^1^Education levels were grouped based on the International Standard Classification of Education (ISCED-2011) codes

^2^ Categories were collapsed due to *N* < 4

^3^ P-values excluding missing categories except for in cohabitation status

A lower percentage of persons with NF1 than persons without NF1 had a long education (34% vs. 60%) and a higher percentage was unemployed (43% vs. 10%). In the group with NF1, the proportions with severe (42%) and moderate disease severity status (25%) as well as severe (66%) and moderate (20%) disease visibility were similar to those reported in the larger questionnaire study (*N* = 244) [18].

### Cognitive functions assessed with performance-based tests

The mean estimated FSIQ was 87.5 (SD = 13.6) for persons with NF1 and 98.8 (SD = 14.6) for the persons without NF1. About 6% of persons with NF1 had an estimated FSIQ < 70 and 46% an estimated FSIQ < 85, whereas none of the comparisons without NF1 had an estimated FSIQ < 70 and 21% had an estimated FSIQ < 85. The MANOVA showed a statistically significant overall difference in cognitive functions between persons with NF1 and the comparisons without NF1, (F(1,141) = 3.905, *p* < 0.0001; Wilks’ lambda = 0.769, partial η^2^ = 0.231). Further, the ANOVAs revealed that persons with NF1 had significantly lower mean estimated FSIQ (zNF1=-0.77; F(1,141) = 18.298, *p* < 0.0001), significantly lower mean immediate visuospatial recall z-score (zNF1=-0.94; F(1,141) = 23.660, *p* < 0.0001), visual short-term memory z-score (zNF1=-0.67; F(1,141) = 9.916, *p* = 0.002), and sustained attention z-score (zNF1=-0.68; F(1,141) = 9.662, *p* = 0.002). No significant differences were observed in mean multitasking, reaction time, motor speed, spatial working memory, planning, or planning time z-scores (Table 2).

**Table 2:** Cognitive functions in adults with NF1 (*N* = 103) and the NF1-free comparison group (*N* = 38)

Cognitive functioning	NF1 *N* = 103			NF1-free controls *N* = 38							
							95% confidence interval		Greenhouse-Geisser		
	Mean raw score (SD)	z-score*	Cohen’s *D*	Mean raw score (SD)	**F based on z-score**	***p-value***	**Lower**	**Upper**	**df**	**F**	***p-value***
Overall									**7.027**	**3.079**	**0.003**
Estimated FSIQ	87.5 (13.6)	-0.77	**0.80**	98.8 (14.6)	18.298	**< 0.0001**	**-0.957**	**-0.587**			
Immediate visuospatial recall	14.9 (7.4)	-0.94	**0.93**	21.7 (7.3)	23.660	**< 0.0001**	**-1.132**	**-0.738**			
Visual short-term memory	5.9 (1.4)	-0.67	**0.61**	6.7 (1.2)	9.916	**0.002**	**-0.890**	**-0.452**			
Multitasking	82.3 (71.4)	-0.21	0.24	65.2 (69.2)	1.635	0.203	-0.453	-0.050			
Reaction time^	--	-0.23	0.26	--	2.339	0.128	-0.392	-0.077			
Motor speed^	--	-0.21	0.18	--	0.747	0.389	-0.450	0.039			
Working memory	13.2 (8.5)	-0.18	0.17	11.7 (8.9)	0.842	0.360	-0.361	0.020			
Planning	9.98 (3.0)	-0.39	0.36	11.0 (2.7)	3.381	0.068	-0.602	-0.171			
Planning time	14.1 (6.7)	0.01	0.01	14.2 (12.6)	0.008	0.931	-0.145	0.123			
Sustained attention	0.86 (0.06)	-0.68	**0.54**	0.89 (0.05)	9.662	**0.002**	**-0.904**	**-0.455**			

SD: standard deviation; FSIQ, full-scale intelligence quotient

*NF1-free comparison cohort as the base, with a mean of 0 and SD = 1

^ No mean raw scores are presented because the subtests were merged by z-scores

The sensitivity analysis revealed slightly worse impairment (as reflected in larger effect sizes (data not shown)) among the persons with NF1 compared to the revised NF1-free control group after exclusion of three participants from that group. However, the same cognitive functions were significantly and not significantly affected, respectively.

### Self-reported executive functions

A MANOVA including the mean Behavioral Regulation Index z-score, the mean Metacognition Index z-score, and the mean Global Executive Composite z-score revealed an overall significant between-group difference (*F*(1,140) = 5.364, *p* = 0.006; Wilks’ lambda = 0.928, partial η^2^ = 0.072). The ANOVAs revealed significant impairments in persons with NF1 compared with persons without NF1 in terms of the Behavioral Regulation Index (BRI) z-score (zNF1=–0.52; *p* = 0.008), the Metacognition Index (MI) z-score (zNF1=–0.61; *p* = 0.002), and the Global Executive Composite (GEC) index z-score (zNF1=–0.63; *p* = 0.001) (Table 3).

**Table 3:** Comparison of self-reported executive functioning domains in adults with NF1 (*N* = 103) and an NF1-free comparison group (*N* = 38)

Executive functioning		NF1 *N* = 103			NF1-free cohort *N* = 38			95% confidence interval	
		Mean raw score (SD)	z- score*	Cohen’s *d*	Mean raw score (SD)	**F based on z- score**	***p-value***	**Lower**	**Upper**
Index	GEC	112.5 (20.0)	-0.63	**0.63**	100.0 (19.5)	10.603	**0.001**	-0.823	-0.428
	MI	65.0 (12.5)	-0.61	**0.61**	57.4 (12.3)	10.342	**0.002**	-0.810	-0.418
	BRI	47.3 (9.3)	-0.52	**0.52**	42.6 (8.9)	7.213	**0.008**	-0.724	-0.323
BRIEF- A subscales	Inhibit	12.3 (2.6)	-0.17	0.21	11.7 (3.1)	1.071	0.303	-0.346	-0.001
	Shift	9.8 (2.5)	-0.66	**0.61**	8.4 (2.1)	9.392	**0.003**	-0.882	-0.440
	Emotional control	16.3 (3.9)	-0.80	**0.71**	13.8 (3.1)	12.075	**0001**	-1.033	-0.562
	Self monitor	9.0 (2.2)	-0.11	0.12	8.7 (2.7)	0.422	0.517	-0.277	0.062
	Initiate	12.7 (3.0)	-0.27	0.27	11.9 (2.9)	1.827	0.179	-0.472	-0.065
	Working memory	14.0 (3.6)	-0.92	**0.86**	11.1 (3.1)	18.675	**< 0.0001**	-1.132	-0.698
	Plan organize	15.9 (3.1)	-0.51	**0.55**	14.1 (3.4)	8.308	**0.005**	-0.687	-0.326
	Task monitor	9.7 (2.1)	-0.49	**0.50**	8.6 (2.3)	7.380	**0.007**	-0.676	-0.305
	Organization of materials	12.7 (3.5)	-0.31	0.31	11.6 (3.6)	2.736	0.100	-0.49	-0.116

SD: Standard deviation; GEC: Global Executive Composite, MI: Metacognition Index, BRI: Behavioral Regulation Index

* NF1-free comparison cohort as the base with a mean of 0 and SD = 1

### Profile of cognitive functions

**Fig. 2:**
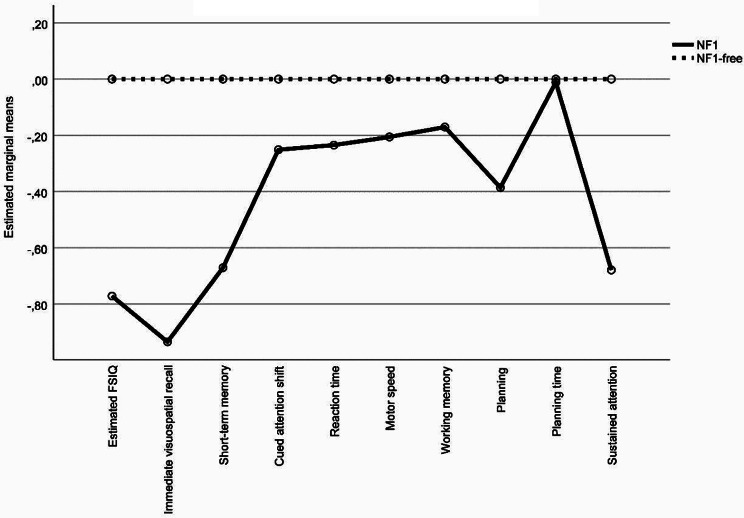
Profile of performance-based cognitive dysfunction in persons with NF1, with NF1-free comparisons as reference

The repeated-measures ANOVA identified a significant group by cognitive functions interaction (*F*(7.027) = 3.079, *p =* 0.003), indicating that some cognitive functions in the persons with NF1 were disproportionally more impaired than others, i.e., revealing a non-flat profile (Table 2; Fig. 2). Subsequent paired-samples *t-*tests further characterized the jagged profile (see supplementary material B).

### Profile of self-reported executive functions

**Fig. 3:**
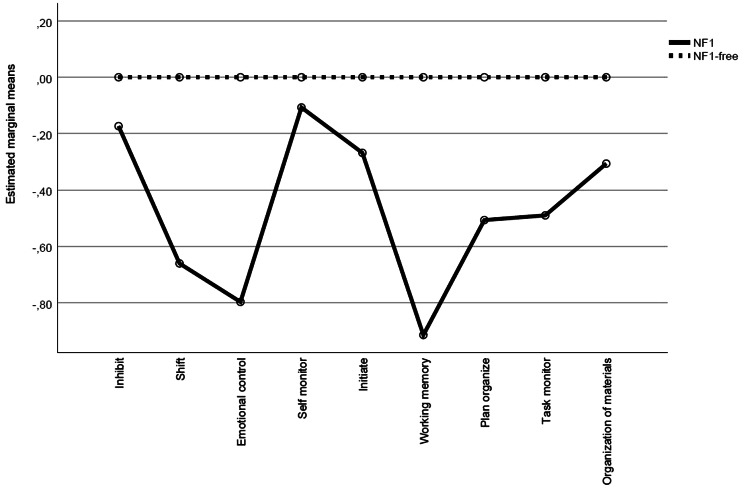
Profile of self-reported executive functioning in persons with NF1, with NF1-free comparisons as reference

A second repeated-measures ANOVA determined a significant group by executive functions interaction (*F*(5.731) = 4.218, *p* < 0.0001), indicating a non-flat executive functioning profile (Fig. 3). Subsequent paired-samples *t-*tests further characterized the jagged profile (see supplementary material B).

## Discussion

To the best of our knowledge, this is the largest neuropsychological study to investigate neurocognitive functioning assessed using both performance-based tests and a self-report measure in a nationwide, population-based sample of adults with NF1 (*N* = 103) and a population-based control sample without NF1 (*N* = 38). We found significantly impaired intelligence, immediate visuospatial recall, visual short-term memory, and sustained attention as well as impairments in the self-reported executive functions in persons with NF1 as compared with population-based controls without NF1. However, multitasking, reaction time, motor speed, spatial working memory, planning time, and planning efficacy as well as some self-reported executive functions in persons with NF1 were not statistically significantly different from those of the population-based controls without NF1. The profile of cognitive functions as well as the profile of self-reported executive functions were both significantly jagged, i.e., some cognitive functions were significantly more impaired than others in persons with NF1. Thus, the neurocognitive functioning of adults with N1 cannot be characterized as global cognitive impairments. Within the profile of cognitive functions, the impairment of intelligence was worse than that of sustained attention and visual short-term memory. Within the profile of executive functions, the impairments of working memory and emotional control were worse than those of e.g. shift, plan-organize, and task monitoring. These impaired, respectively, relatively spared cognitive functions contribute to a detailed cognitive functioning profile of NF1 in adults, and the impaired cognitive functions may reflect the pathophysiology of NF1.

Our findings confirm and extend previous research of lowered mean intelligence in persons with NF1. The mean estimated FSIQ of 87.5 (SD = 13.7) among persons with NF1 was similar to the mean FSIQs reported in previous studies. A downward shift in FSIQ of approximately 10 IQ points in clinical NF1 samples when compared to siblings or age, gender, and socioeconomically matched comparisons has been observed [6, 14, 28, 28]. The observed prevalence of intellectual disability (FSIQ < 70) [29] in persons with NF1 was 6% which is similar to the result of Ferner et al. (1996), who found 8% of children and adults with NF1 to have an FSIQ < 70 [14]. Pavol et al. (2006) suggested that perhaps persons with NF1 do not present with a profile of severe cognitive deficits because the widespread neuronal abnormalities occur very early in development, allowing the brain to adapt to, but not fully overcome the genetically imposed impairments [12].

Our findings of impaired immediate visuospatial recall, with a large effect, as well as visual short-term memory with a moderate effect are in line with prior findings [10, 13]. Memory is essential for many aspects of real-life functioning, such as academic performance and everyday life activities, and memory deficits may strongly reduce quality of life, as reported in a population-based study in the elderly [30].

We found no significant difference in motor speed between persons with and without NF1, confirming a previous finding in psychiatrically healthy adults with NF1 [5]. In terms of sustained attention, we observed significant impairments in persons with NF1, which contrasts an earlier finding in psychiatrically healthy adults with NF1 [5] and in adults with NF1 and no psychiatric comorbidity except ADHD [8]. Hyman et al. (2006) found that only 35% of children with NF1 who fulfilled the criteria for ADHD had received a clinical diagnosis of ADHD underlining the need for clinical focus on ADHD in the NF1 population [31].

Our results showed that persons with NF1 have somewhat similar reaction times (readiness to respond to a given stimulus or event) as persons without NF1. These finding contrasts earlier findings of significantly slower reaction times in subjects with NF1 e.g. in a study of adolescents [17], in adults [13], and in a NF1 sample with a wide age range of 6–75 years [14]. Further studies should be conducted to characterize reaction time in adults with NF1.

Considering only the performance-based test results, no aspects of executive functioning (spatial working memory, planning efficacy, or planning time) were significantly impaired in our results on persons with NF1 as compared with persons without NF1. These findings are generally in line with those of Descheemaeker et al. (2013) [10]. In terms of working memory, our observation is in line with an earlier report of a non-significant difference between adults with NF1 and controls [13], but it contrasts the working memory impairments found in adolescents with NF1 when compared to matched controls [17]. We found no significant impairment in multitasking, which may be surprising given the high prevalence of Autism Spectrum Disorder (ASD) in persons with NF1 [32]. Persons with ASD have been found having impaired multitasking [33, 34] where executive problems of planning inflexibility, inhibition, and difficulties with prospective memory may lie behind [34].

In contrast to our performance-based executive functioning results, we observed several significantly impaired self-reported executive functions in daily life among persons with NF1 as compared with persons without NF1. Participants with NF1 reported significant impairments in several aspects of executive functions, including planning/organization and working memory. The discrepancy between performance-based and self-reported executive functions in terms of working memory and planning may be related to measurement differences. In the performance-based tests, we applied executive tasks demanding responses to single events in a quiet, structured, standardized testing environment, while the self-reported measure includes questions addressing real-life situations in which persons with NF1 may have difficulties. Performance based test results may reflect optimal executive function capacity [35], however, to depict a more realistic functioning, self- or proxy-ratings are useful due to the more natural setting and the capturing of a greater window of behavior [36]. The self-reported questionnaire-based impairments of executive functions such as mental flexibility, emotional control, working memory, task monitoring, and planning and organizing may contribute to impairments of everyday life functioning and may partly explain why 30% of persons with NF1 in our sample receive social security benefits and only 13% have a full-time job. Such potentially important executive functioning predictors will be examined in the next step of the study.

### Strengths and limitations

Our study is the first nationwide population-based study to characterize a profile across neurocognitive domains and a separate profile within the executive functioning domain with the largest sample size of adults with NF1. Our sample represents 24% of the total Danish population of adults diagnosed with NF1 from 1977 onwards and the NF1-related characteristics such as disease severity and visibility are similar to those of the participants in the prior and much larger questionnaire study [18]. Still, we may not have reached the least resourceful adults with NF1, which may imply underestimation of the cognitive burden. Application of a comprehensive neurocognitive assessment, including both performance-based neuropsychological tests and self-reported questionnaires is a strength, allowing both a comprehensive objective and a self-reported questionnaire-based description of cognitive functioning.

The results of this study need to be considered in the context of its limitations, and several issues are still awaiting further exploration. We did not reach the size of the comparison group (*N* = 38) that we had aimed for (*N* = 50). However, a post-hoc power estimation showed that with an expected difference in FSIQ of approximately 10 points [6, 14], a sampling ratio of 3:1 with 99 persons with NF1 and 33 NF1-free persons, and a standard deviation of 14 [6, 14], the power to detect a difference of 10 points is 0.94. Concerning this main outcome, the sample size and unequal sampling ratio does not seem to be an issue. Still, we cannot exclude that some of the non-significant differences (e.g. the performance-based planning score) were due to lack of power. We used a population-based NF1-free sample to examine cognitive functioning in NF1, and our results reflect cognitive impairments in NF1 beyond what is observed in the general population and may explain the less severe cognitive impairments observed in our study compared to previous studies of convenient samples.

We did consider age and gender as potential confounders and thus matched NF1-free persons on these characteristics and did not observe between group differences on these. We did observe significant differences in education between NF1 and NF1-free groups, which we hypothesize are partially related to NF1. We carefully considered adjusting for FSIQ and education, which are both associated with NF1 [37, 38]. However, as we expected intelligence to be a mediator rather than confounder in a causal pathway between NF1 status and cognitive function, and as we expected education to be a consequence of executive function, we thus decided not to match on them neither to include them in the models [39]. No formal calibration of potential tester differences was performed among the 10 psychology students. However, a main part of the tests was performed through the automated CANTAB system which has been developed specifically to uniform the standardized test procedure. Furthermore, to minimize tester variations, all testers underwent a four-day training program including a final assessment of alignment with the test manual, which they had to pass before test initiation. Finally, we did not include a performance-based measure of social cognition. As it has been established that adults with NF1 may struggle with social life, further research is needed to examine if this may be related to impairments in social cognition. Overall, there is a lack of studies on how the cognitive impairment may impact the real-life functioning such as participation in social and work life.

Identification of cognitive impairment is a starting point for enabling persons with NF1 in seeking and receiving support: Our results suggest that neuropsychological assessment guidelines using a standard battery of neuropsychological tests and questionnaires should be developed to more systematically characterize cognitive functioning in adults with NF1 in the clinic. Furthermore, systematic information on cognitive functioning could enable research scaled to develop interventions to manage impaired cognitive functions in adults with NF1 with a large potential for improving quality of life.

## Conclusion

This study offers new insight into the cognitive functioning profile of adults with NF1. Performance-based measures revealed a pattern of significant impairments of intelligence, immediate visuospatial recall, and sustained attention while a self-report questionnaire revealed impairments in several aspects of executive functions. Neither the cognitive profile nor the executive functions profile of NF1 showed generalized impairments. Our results underline the importance of attention to the cognitive functioning of adults with NF1 in the clinic.

## Electronic supplementary material

Below is the link to the electronic supplementary material.

Supplementary Material 1

Supplementary Material 2

## Data Availability

The participants of this study did not give written consent for their data to be shared publicly, so due to the sensitive nature of the research data is not available.
