# Supplementary material for: Neurocognitive functioning in adults with neurofibromatosis type 1- a nationwide population-based study
Source: Orphanet J Rare Dis. 2024 Nov 28;19:441. doi: 10.1186/s13023-024-03454-w (PMC11603635; doi:10.1186/s13023-024-03454-w)
Supplement: Supplementary file 2 — Supplementary Material 2 [file 13023_2024_3454_MOESM2_ESM.docx]

**Supplementary material A**

**Wechsler Adult Intelligence Scale (WAIS), Fourth Edition**

We used an abbreviated version of the WAIS-IV (1) to assess intelligence as indexed by a full-scale intelligence quotient (FSIQ) estimate. Reliable estimates for intelligence have been shown with only four or seven selected subtests (2). We used the four subtests Vocabulary, Similarities, Block design, and Matrix reasoning and Danish norms were used to estimate the FSIQ (3).

*Vocabulary* is a subtest for word knowledge and verbal ability. Words are presented visually and orally. This test requires receptive and expressive vocabulary, but no motor response. The participant must only understand that he/she needs to define the words presented, where an appropriate “definition” can also be a single word (synonym) (3).

*Similarities* is a subtest that measures language conceptualization, verbal abstraction, and analogical verbal reasoning. The test examines abstract thinking and finding similarities among words or ideas that may appear dissimilar. This test also requires receptive language skills and moderate demand for expressive language skills. No visual information is presented, and no motor response is required (3).

The *Block design* is a visual and motor test measuring the ability to recognize geometric formations, analyse visual information, and recreate the geometric design shown, using six-sided blocks. This is a timed task and places demand primarily on spatial visualization ability and motor skills employing the 1-inch blocks. Block design desires to break down patterns from larger parts into smaller parts and reverse. This task can demonstrate an individual’s approach to problem-solving and is consequently associated with intelligence (3).

*The Matrix reasoning* is a subtest, presented visually to measure “non-verbal abstract problem-solving, inductive reasoning, and spatial reasoning” (3). The task demands “pattern recognition, attention to visual details, including shape, color, pattern, and location, and recognition of the relationship between parts/features” (3). Minimal demands are placed on receptive language, and no requirement is made for expressive language (3).

**The Cambridge Neuropsychological Test Automated Battery (CANTAB)**

Cognitive functions in several domains were assessed with CANTAB subtests (4-5) in a computerized neuropsychological test battery developed with a neuroscience approach. The validity and reliability of CANTAB tests have been demonstrated for persons aged 4–80 years (6-10). The CANTAB tests were presented on a tablet with a touch screen placed in front of the tested person. The Motor Screening Test (MOT) screens for potential difficulties in basic visual, motor, and task comprehension. Participants were asked to touch a cross appearing at various places on the screen by time. For this study, we applied the test as an initial screen for the ability to perform further CANTAB subtests. The outcome measure covers the number of trials, on which the participant responded correctly. All participants reached full scores on a number of correct responses (data not shown) and therefore proceeded with further subtests. For the present study, the CANTAB subtests included were the following (see variables in supplementary Table A):

*Multitasking:* The Multitasking Test (MTT) assesses cued attentional set-shifting between tasks. Response time, as well as the errors in cued switching trials are measured over a number of trials. For each trial, an arrow appeared right or left on the screen. Further, a cue was shown within each trial, stating if the participant has to point on the direction of the arrow, or the screen-side where the arrow appeared. Some trials are designed incongruently. Outcome measures covered incongruency cost (the difference between the median latency of response on the trails that were congruent versus incongruent) as well as multitasking cost (the difference between the median latency of response during assessed blocks in which both rules were used versus only one rule was used) (4, 11).

*Planning and Planning time:* The One-touch Stocking of Cambridge (OTS) is an executive function test of planning. Participants estimated the minimum number of moves it would take to rearrange a set of balls in pockets shown on the lower part of the screen to reproduce the arrangement on the upper part of the screen. They indicated the estimated minimum number of moves by choosing from a set of numbers on the screen (12). The number of problems solved with the first attempt and the median latency time to the first correct attempt were key outcome measures.

*Working memory:* The Spatial Working Memory (SWM) test measures working memory for spatial stimuli. An array of boxes is presented, and participants are asked to search through the boxes for hidden tokens. One box at a time was touched until a blue token was found inside. The token was then placed ‘home’ on the right side of the screen (13). Participants then made “a new search for the next token”. The key instruction was that, once a token had been located, that box would not be used again to hide another token. This was repeated until a token had been located in each box. After four practice trials with three boxes (13), four experimental trials were run with each of four, six, and eight boxes (13). The task places an eminent demand on central executive functioning by requiring subsequent searches to be limited to boxes that had not yet yielded a token (14). Outcome measures include numbers of errors and strategy, quantified by the number of distinct boxes used to begin a new search for a token for the same problem.

*Visual short-term memory:* The Spatial Span (SSP) test measures visual short-term memory (15). Participants remembered a sequence of two or more squares on the screen that changed color one by one. Trials progressed from two to nine squares, and the test self-terminated after three successive failures on a given number of squares (16,17). The outcome measure states the maximum number of squares remembered in the correct order.

*Sustained attention:* The Rapid Visual Information Processing (RVP) test assesses sustained attention. In the center of the screen, a white box appeared with digits 2 – 9 in a pseudo‐random order with 100 digits per minute. After short training, participants took a four‐minute assessment in which they were screened for the sequences 2‐4‐6, 3‐5‐7, and 4‐6‐8 and should push a button, as soon as the targeted sequences was recognized. The formal definition of the key RVP outcome variable A´ is stated as being complex, including the difference between the probability of hit and the likelihood probability of false alarm (18). With this test, the number of responses recorded in 1,800 ms of the final digit presentation for each sequence are calculated (19, 20,11).

*Reaction time and motor speed:* The Reaction Time (RTI) test evaluates movement time and reaction time. “The test includes simple and five-choice reaction time segments and distinguishes between reaction time and movement latencies. The reaction time is the speed with which the participant releases the pressed key in response to the onset of a stimulus. The movement time (motor speed) represented by the time taken to touch the stimulus on the screen after the pressed key has been released” (21). Outcomes are measured in milliseconds (21).

*Immediate visuospatial recall*: The Rey´s Complex Figure Task (RCFT) permits observation of visual-spatial memory (22). Participants are instructed to copy the complex figure as accurately as possible without rotating the stimulus or the sheet of paper. After 3 minutes, and without mentioning a forehead, the person is instructed to recall the figure and produce a second drawing. Inspired by the terminology of manual, the performance on this “3-minute Immediate Recall trial” (p. 3) was reflective of immediate visuospatial recall.

*Self-reported executive functions in everyday life:* We used the Danish self-report version of the Behavior Rating Inventory of Executive Function – Adult Version (BRIEF-A) to assess executive functions in everyday life (23). The BRIEF-A is designed to be completed by adults aged 18–90 years with a minimum (5th-grade school) reading level. The BRIEF-A consists of 75 items in nine subscales for measuring different aspects of executive functioning: Inhibit, Shift (ability to alternate or switch attention), Emotional control, Self-monitor, Initiate, Working Memory, Plan/Organize, Task monitor (ability to assess one’s own performance), and Organization of Materials. Each item was rated based on a Likert scale from 1 = never, 2 = sometimes, and 3 = often (23). The Global Executive Composite (GEC), which is the overall summary score, and two clinical indexes, the Behavioral Regulation Index (BRI) and the Metacognition Index (MI), were computed. BRIEF-A has not been formally validated in Denmark. However, the Danish version of the BRIEF-Adult version is generally accepted and was e.g. recently included in a study on cognitive dysfunctions after cardiac arrest (24).

**Supplementary references**

1. Wechsler, D. WAIS-IV, Wechsler Adult Intelligence Scale. Fourth Edition. Dansk Version. Enschede: NCS Pearson Inc.; 2011.
2. Meyers JE, Zellinger MM, Kockler T, Wagner M, Miller RM. (2013). A validated seven-subtest short form for the WAIS-IV. Applied Neuropsychology – Adult. 2013; 20(4): 249–256.
3. Wechsler, D. Wechsler Adult Intelligence Scale. Fourth Edition. San Antonio, TX: NCS Pearson Inc.;2008.
4. Cognition Cambridge Ltd. Cambridge neuropsychological test automated battery. Cambridge: Cambridge Cognition Ltd.; 1996.
5. CANTAB® (Cognitive assessment software). Cambridge Cognition All rights

reserved. [www.cantab.com](http://www.cantab.com); 2019.

1. De Luca CR, Wood SJ, Anderson V, Buchanan JA, Proffitt TM, Mahony K, Pantelis C. Normative data from the CANTAB. I: Development of executive function over the lifespan. Journal of Clinical and Experimental Neuropsychology. 2003; 25(2): 242–254.
2. Housden C, Hermans L, Barnett J, Cormack F, Blackwell A, Abbott R. Validation of an automated cognitive assessment instrument (Abstract C10). Journal of Psychopharmacology. 2015; 29(Suppl.):A62.
3. Kim H S, An YM, Kwon JS, Shin MS. A preliminary validity study of the Cambridge neuropsychological test automated battery for the assessment of executive function in schizophrenia and bipolar disorder. Psychiatry Investigation. 2014;11(4): 394–401.
4. Robbins TW, James M, Owen AM, Sahakian BJ, McInnes L, Rabbitt P. Cambridge neuropsychological test automated battery (CANTAB): A factor analytic study of a large sample of normal elderly volunteers. Dementia. 1994; 5(5): 266–281.
5. Smith P, Need A, Cirulli E, Chiba-Falek O, Attix D. A comparison of the Cambridge Automated Neuropsychological Test Battery (CANTAB) with “traditional” neuropsychological testing instruments. Journal of Clinical and Experimental Neuropsychology. 2013; 35(5): 319–328.
6. Khassawneh BY, Bathgate CJ, Tsai SC, Edinger JD. Neurocognitive performance in insomnia disorder: The impact of hyperarousal and short sleep duration. Journal of Sleep Research. 2018; 27(6): 1–10.
7. Chamberlain SR, Derbyshire KL, Leppink EW, Grant JE. Neurocognitive deficits associated with antisocial personality disorder in non-treatment-seeking young adults. Journal of the American Academy of Psychiatry and the Law. 2016; 44: 218–225.
8. Payne JM, Arnold SS, Pride NA, North KN. Does attention-deficit-hyperactivity disorder exacerbate executive dysfunction in children with neurofibromatosis type 1? Developmental Medicine and Child Neurology. 2012; 54(10): 898–904.
9. Baddeley A, Logie R. Working memory: The multiple-component model. In: A. Miyake & P. Shah (eds), Models of Working Memory (pp. 28–61). Cambridge: Cambridge University Press; 1999.
10. Teixeira RAA, Zachi EC, Roque DT, Taub A, Ventura DF. Memory span measured by the spatial span tests of the Cambridge Neuropsychological Test Automated Battery in a group of Brazilian children and adolescents. Dementia & Neuropsychologia. 2011; 5(2): 129–134.
11. Bourke C, Porter R J, Carter JD, McIntosh VV, Jordan J, Bell C, Carter F, Colhoun H, Joyce PR. Comparison of neuropsychological functioning and emotional processing in major depression and social anxiety disorder subjects, and matched healthy controls. Australian and New Zealand Journal of Psychiatry. 2012; 46(10): 972–981.
12. Racinais S, Gaoua N, Grantham J. Hyperthermia impairs short-term memory and peripheral motor drive transmission. Journal of Physiology. 2008;19: 4751–4762.
13. Elliott R, Sahakian BJ, Matthews K, Bannerjea A, Rimmer J, Robbins TW. Effects of methylphenidate on spatial working memory and planning in healthy young adults. Psychopharmacology. 1997;131(2): 196–206.
14. Coull JT, Frith CD, Frackowiak R.SJ, Grasby PM. Modulation of attentional networks by the α2 adrenoceptor agonist clonidine: limitations on experimental design. NeuroImage. 1996; 3(3): S177.
15. Gau SS, Huang W. Rapid visual information processing as a cognitive endophenotype of attention deficit hyperactivity disorder. Psychological Medicine. 2014; 435–446.
16. Pereira T, Cipriano I, Costa T, Saraiva M, Martins A. Exercise, ageing and cognitive function – Effects of a personalized physical exercise program in the cognitive function of older adults. Physiology and Behavior. 2019; 202: 8–13.
17. Meyers JE, Meyers KR. Rey Complex Figure Test and Recognition Trial: Professional Manual. Lutz, FL: Psychological Assessment Resources Ltd.; 1995.
18. Roth RM, Isquith PK, Gioia GA. BRIEF-A: Behavior Rating Inventory of Executive Function –Adult Version. Lutz, FL: Psychological Assessment Resources Ltd.; 2005.
19. Jensen MK, Christensen J, Zarifkar P, Thygesen LC, Wieghorst A, Berg SK, Hassager C, Stenbæk DS, Wagner MK. Evaluating neurocognitive outcomes in out-of-hospital cardiac arrest survivors: A comparative study of performance-based and reported measures. Resuscitation. 2024;202:110310.

**Supplementary Material B**

***Performance-based cognitive functioning profile***

Subsequent paired-sample *t-*tests demonstrated a jagged profile, with the mean estimated FSIQ z-score significantly lower than those for reaction time (t=–3.89, df=102, *p*=0.0002), motor speed (t=–3.28, df=102, *p=*0.0013), working memory (t=–4.34, df=102, *p*<0.0001), and planning time (t=–5.13, df=102, *p*<0.0001).

The mean immediate visuospatial recall z-score was also significantly lower than those for multitasking (t=–4.36, df=102, *p*<0.0001), reaction time (t=–5.22, df=102 *p*<0.0001), motor speed (t=–4.31, df=102, *p*<0.0001), planning (t=–3.79, df=102, *p*=0.0002), working memory (t=–5.72, df=102, *p<*0.0001), and planning time (t=–5.76, df=102, *p*<0.0001).

Visual short-term memory was significantly lower than working memory (t=–3.78, df=102, *p*=0.0002) and planning time (t=–3.73, df=102, *p*=0.0003). Reaction time (t=3.34, df=102, *p*=0.0011), working memory (t=3.58, df=102, *p*=0.0005), and planning time (t=3.83, df=102, *p*=0.0002) were significantly higher than sustained attention.

None of the other comparisons were significantly different.

***Patient-reported executive functioning profile***

A subsequent paired-samples *t-*test characterized the jagged profile, showing that the mean z-scores in the subscale Shift were significant worse than those in the subscales Inhibit (t=–4.771, df=102, *p*<0.0001), Self-monitor (t=–5.054, df=102, *p*<0.0001), and Initiate (t=–3.827, df=102, *p*<0.0002).

The mean z-score of the subscale Emotional Control was significantly worse than those of the subscales Self-monitor (t=–6.147, df=102, *p*=0.0001), Initiate (t=–3.911, df=102, *p*=0.0002), and Organization of Materials (t=–3.56 df=102, *p*=0.0006).

The mean z-score of the subscale Working Memory was significantly worse than those of the subscales Inhibit (t=–7.397, df=102, *p*=0.0001), Self-monitor (t=–8.041, df=102, *p*<0.0001), Initiate (t=–6.781, df=102, *p*<0.0001), Plan/organize (t=–4.754, df=102, *p*<0.0001), Task Monitor (t=–5.125, df=102, *p*=0.0001), and Organization of Materials (t=–5.15, df=102, *p*=0.0001).

**Supplementary Table A: Overview of all measures, with the instrument and cognitive domain**

|  | **Neuropsychological task** | **Instrument** | | **Main outcome measures** | **Merged variables and domains** |
| --- | --- | --- | --- | --- | --- |
| **Performance-based measures** | Visuospatial memory,  visual long-term memory | **RCFT** | Recall | Immediate recall (3 min) |  |
|  | Intelligence | **WAIS-IV** | Matrix reasoning | Number of correct tasks | Estimated Full-scale Intelligence Quotient (FSIQ) |
|  |  |  | Block design | Solved puzzles |  |
|  |  |  | Similarities | Sum score for correct items |  |
|  |  |  | Vocabulary | Sum score for correct items |  |
|  | Working memory | **CANTAB** | Spatial Working Memory (SWM) | Total error  Strategy | Total error selected for “working memory” |
|  | Sustained attention,  information processing |  | Rapid Visual Information Processing (RVP) | Sensitivity to target sequence | RVPA’ = “sustained attention” |
|  | Multitasking |  | Multi-tasking Test (MTT) | Incongruency cost Multi-tasking cost | Multi-tasking and Incongruency cost merged into “Cued attentional set shift” |
|  | Mental response speed,  reaction time  Processing speed |  | Reaction Time Task (RTI) | Simple Median Reaction Time  Median Five Choice Reaction Time Simple Movement time Median Five Choice Movement Time | Simple Median and Five Choice Reaction time merged into “Reaction time”  Simple Median and Median Five Choice Movement time merged into “Motor Speed” |
|  | Spatial planning,  Latency, planning time |  | One Touch Stockings of Cambridge (OTS) | Problem Solved to First Choice  Median Latency to First Choice | Problem Solved to First Choice = Planning  Median latency to First Choice = “Planning time” |
|  | Short-term spatial memory |  | Spatial Span (SSP) | Forward Span Length | SSPFSL = “visual short term memory” |
| **Self-reported questionnaire** | Executive functions | **BRIEF-A** | Global Executive Composite (total score)  Metacognition Index (MI)  Behavioral regulation Index (BRI) | BRI: Inhibit, shift, emotional control, self-monitor  MI: initiate, working memory, plan organize, task monitor, organization of materials | GEC  MI  BRI |

RCFT: Rey’s complex figure task; WAIS-IV: Wechsler Adult Intelligence Scale; CANTAB: Cambridge Neuropsychological Test Automated Battery; BRIEF-A: Behavior Rating Inventory of Executive Functioning – Adults

**Supplementary Table B: Outcomes in adults with NF1 and an NF1-free comparison group**

| **Test** |  | **NF1**  **N = 103** |  | **NF1-free group**  **N = 38** | **NF1 N = 103** | | **NF1-free N = 38** |  |  |
| --- | --- | --- | --- | --- | --- | --- | --- | --- | --- |
|  |  |  |  |  |  | |  | **95% confidence interval** | |
|  |  | Mean raw score (SD) | **z- score*** | Mean raw score (SD) | **F based on z- score** | | ***p-value*** | **Lower** | **Upper** |
| **Wechsler Adult Intelligence Scale IV** | | |  |  |  | |  |  |  |
| Estimated full-scale intelligence quotient (FSIQ) | Block design, Matrix reasoning, Similarities, Vocabulary | 87.5 (13.7) | -0.77 | 98.8 (14.6) | 17.175 | **< 0.0001** | | **-0.571** | **-0.945** |
| **Rey’s Complex Figure Task** | | |  |  |  | |  |  |  |
| Visual long-term memory | Recall after 5 min | 14.9 (7.5) | -0.94 | 21.7 (7.3) | 22.209 | **< 0.0001** | | **-0.727** | **-1.129** |
| **CANTAB** |  |  |  |  |  | |  |  |  |
| Spatial Working Memory (SWM) | Total error  Strategy | 13.2 (8.5) | -0.18 | 11.7 (8.9) | 0.892 | | 0.347 | -0.376 | 0.15 |
|  |  | 7.6 (2.5) | 0.17 | 8.0 (2.5) | 0.739 | | 0.391 | -0.31 | 0.364 |
| Rapid Visual Information Processing (RVP) | Sensitivity to target sequence | 0.86 (0.06) | -0.68 | 0.89 (0.05) | 7.943 | | **0.006** | **-0.394** | **-0.843** |
| Multi-tasking Test (MTT) | Incongruency cost | 82.3 (71.4) | -0.21 | 65.2 (69.2) | 1.030 | | 0.312 | -0.412 | 0.001 |
|  | Multitasking cost | 301.0 (164.5) | -0.22 | 289.8 (149.9) | 0.956 | | 0.330 | -0.446 | 0.010 |
| Reaction Time Task (RTI) | Median simple Reaction time | 365.5 (40.8) | -0.18 | 355.4 (58.4) | 1.648 | | 0.201 | -0.343 | -0.039 |
|  | Median five choice Reaction time | 409.8 (46.8) | -0.31 | 393.6 (57.7) | 3.320 | | 0.071 | -0.483 | -0.136 |
|  | Median Simple movement time | 252.8 (122.4) | -0.17 | 242.8 (95.0) | 0.422 | | 0.517 | -0.428 | 0.095 |
|  | Median five choice Movement time | 293.5 (128.7) | -0.30 | 265.4 (95.2) | 1.550 | | 0.215 | -0.544 | -0.054 |
| One Touch Stocking of Cambridge (OTS) | Problem solved on first trial | 9.98 (3.0) | -0.39 | 11.0 (2.7) | 3.405 | | 0.067 | -0.177 | -0.615 |
|  | Median latency on first trial | 14.1 (6.7) | -0.01 | 14.2 (12.7) | 0.011 | | 0.918 | -0.150 | 0.122 |
| Spatial span (SSP) | Forward span length | 5.9 (1.4) | -0.67 | 6.7 (1.2) | 8.436 | | **0.004** | **-0.406** | **-0.846** |

SD: Standard deviation, CANTAB: Cambridge Neuropsychological Test Automated Battery

* NF1-free comparison cohort as the base with a mean of 0 and SD = 1
